# Effects of hydromorphone for patient-controlled intravenous analgesia on postpartum depression and sleep disorders after cesarean section: a randomized, double-blind controlled trial

**DOI:** 10.3389/fphar.2026.1802702

**Published:** 2026-04-24

**Authors:** Yun Zhu, Chao Wang, Xiangxiang Chen, Yuanyuan Zhao, Lei Yang, Qi Wang, Jie Lv, Wei Wang

**Affiliations:** 1 Department of Anesthesiology, Nanjing Jiangning Hospital of Traditional Chinese Medicine, Nanjing, China; 2 Department of Anesthesiology, The Affiliated Jiangning Hospital of Nanjing Medical University, Nanjing, China; 3 Department of Anesthesiology, Huainan First People’s Hospital, The First Affiliated Hospital of Anhui University of Science and Technology, Huainan, China

**Keywords:** cesarean section, hydromorphone, patient-controlled intravenous analgesia, postpartum depression, sleep disorders

## Abstract

**Background:**

Postpartum depression (PPD) is a significant concern in obstetrics. This study aimed to investigate the effects of hydromorphone in patient-controlled intravenous analgesia (PCIA) on PPD and sleep disorders following cesarean section.

**Methods:**

A total of 140 parturients undergoing elective cesarean section under combined spinal-epidural anesthesia, with singleton full-term pregnancy and aged 22–35 years, were enrolled from July to December 2025 at Jiangning Hospital Affiliated to Nanjing Medical University. Using a random number table, participants were allocated to two groups based on PCIA formulation: the sufentanil group (Group S) received sufentanil (3 μg/kg) + tropisetron (0.1 mg/kg) diluted to 150 mL with normal saline; the hydromorphone group (Group H) received hydromorphone (0.15 mg/kg) + tropisetron (0.1 mg/kg) diluted to 150 mL with normal saline. The primary outcome was the incidence of PPD at 6 weeks postoperatively. Secondary outcomes included PPD incidence at 1 week, Ramsay sedation scores at 24 and 48 h, Pittsburgh Sleep Quality Index (PSQI) scores preoperatively and at 24/48 h, resting Visual Analog Scale (VAS) pain scores at 6, 12, 24, and 48 h, PCIA button presses and rescue analgesia within 48 h, cumulative 48-hour opioid consumption, bowel function recovery time, first lactation time, neonatal behavioral neurological assessment (NBNA) scores at 48 h postoperatively and adverse reactions.

**Results:**

The incidence of PPD at 6 weeks was significantly lower in Group H compared to Group S [5.8% vs. 20.6%, relative risk (RR) = 0.28, 95% confidence interval (CI) 0.10 to 0.78, *P* = 0.004]. The absolute risk reduction was 14.8% (95% CI 4.2%–25.4%), corresponding to a number needed to treat (NNT) of 7 (95% CI 4–24). Ramsay and PSQI scores at 24 and 48 h were also significantly lower in Group H (*P* < 0.01 for all), as was the incidence of dizziness and drowsiness within 48 h (*P* < 0.01). No significant intergroup differences were found in analgesia-related outcomes or other adverse reactions.

**Conclusion:**

Hydromorphone for PCIA after cesarean section can reduce the incidence of PPD at 6 weeks, improve early postoperative sleep quality, and decrease dizziness and drowsiness.

**Clinical Trial Registration:**

https://www.chictr.org.cn/bin/home, identifier ChiCTR2500105264.

## Introduction

In recent years, adverse events such as maternal suicide and infanticide due to postpartum depression (PPD) have occurred from time to time, drawing increasing attention to the mental health of perinatal women. Postpartum depression (PPD) is a common psychiatric disorder in obstetrics, characterized by depressive mood and related symptoms during the puerperium (Liu et al., 2026). Its clinical manifestations include anxiety, irritability, agitation, fear, depression, and impaired adaptation to stressors (Alkhateeb et al., 2026). PPD can occur following both vaginal and cesarean deliveries (Froeliger et al., 2026). The first six weeks postpartum represent a high-risk period for PPD after cesarean section, with an estimated incidence ranging from 4% to 40% (Froeliger et al., 2026; Pe et al., 2026). Etiology is multifactorial, with major risk factors including perinatal pain, preexisting depressive mood, poor sleep quality, family discord, and comorbidities (Xie et al., 2026; Redhead et al., 2026). PPD adversely affects maternal health, breastfeeding, and family wellbeing. Current treatment primarily involves pharmacotherapy combined with psychotherapy, but long-term medication use may negatively impact infant development via breastfeeding (Clark et al., 2026; Galbally et al., 2026). Therefore, prevention is paramount.

For women undergoing cesarean section, postoperative PCIA effectively manages pain. However, commonly used opioids like sufentanil lack antidepressant properties and are associated with adverse effects such as nausea, vomiting, dizziness, and drowsiness (Sutton and Carvalho, 2017). Hydromorphone is a semi-synthetic morphine derivative. Its chemical structure, featuring a ketone group at the 6-position, confers greater analgesic potency and reduced metabolic accumulation compared to morphine (Meissner et al., 2023; Rodrigues et al., 2021). Studies suggest hydromorphone for postoperative analgesia can improve sleep quality in gynecological patients and reduce depression scores in orthopedic patients (Ma et al., 2022; Wang et al., 2025). We therefore hypothesized that hydromorphone-based PCIA after cesarean section might be associated with a reduced incidence of PPD and alleviated sleep disturbances, possibly through its distinct pharmacological profile and improved early recovery experience. This study aimed to test this hypothesis.

## Methods

### Ethics and registration

This study adhered to the Consolidated Standards of Reporting Trials (CONSORT) guidelines (Hopewell et al., 2025). Ethical approval (No. 2025-03-076-H01) was obtained from the Ethics Committee of the Affiliated Jiangning Hospital of Nanjing Medical University. The trial was registered at the Chinese Clinical Trial Registry (ChiCTR2500105264) on July 1, 2025. All participants provided written informed consent.

### Participants

Primiparas scheduled for elective cesarean section from July to December 2025 were recruited. Inclusion criteria were: singleton full-term pregnancy, American Society of Anesthesiologists (ASA) physical status II, age 22–35 years, and BMI 22–32 kg/m^2^. Exclusion criteria included: history of psychiatric disorders or intellectual disability; preoperative organic or drug-induced depression; abnormal fetal position; pregnancy complications (e.g., hypertension, diabetes); major organ dysfunction; chronic sleep disorders or preoperative PSQI >5 (Shaowei et al., 2026); preoperative Edinburgh Postnatal Depression Scale (EPDS) score >9 (Roddy et al., 2026). Intraoperative exclusion criteria were: failed neuraxial anesthesia requiring conversion; sensory block level (by temperature) above T4 or inadequate for surgery requiring epidural supplementation; additional surgical procedures (e.g., cystectomy); surgery duration >2 h; blood loss >500 mL.

### Sample size calculation

Sample size calculation was based on a pilot study of 20 participants (10 per group), which showed a PPD incidence at 6 weeks of 20% in the sufentanil group and 5% in the hydromorphone group, corresponding to a 15% absolute risk reduction. To detect this difference with a two-sided α = 0.05 and power (1-β) = 0.8 using a χ^2^ test, a sample size of 126 participants was required (calculated using PASS software). Assuming a 10% dropout/loss to follow-up rate, we aimed to enroll 140 participants (70 per group). The primary endpoint (PPD incidence at 6 weeks) was prespecified in the trial registration.

### Randomization and blinding

Participants were randomly assigned using a sequence generated by SPSS 26.0. An independent statistician prepared sequentially numbered, sealed, opaque envelopes. Participants, outcome assessors, and data analysts were blinded. An anesthesiologist not involved in the study opened the envelope prior to PCIA initiation to prepare the PCIA pump.

### Interventions

All parturients underwent routine preoperative fasting for 8 h and fluid restriction for 2 h, with no preoperative medication administered. After entering the operating room, routine monitoring of electrocardiography (ECG), heart rate (HR), non-invasive blood pressure (NIBP) and pulse oxygen saturation (SpO_2_), was established, and oxygen was administered via face mask. All parturients received combined spinal-epidural anesthesia, with the puncture performed at the L_3_ - L_4_ interspace. For the subarachnoid block, 8–10 mg of 0.5% bupivacaine was administered, injected within 20 s. An epidural catheter was inserted cephalad for potential supplementation. Ten minutes later, the sensory block level (tested by pinprick or cold sensation) was determined. Patients were excluded from the study if the level was too high (above T_4_) or too low (insufficient for surgery, requiring supplemental local anesthetic via the epidural catheter). All anesthetic procedures were performed by the same anesthesiologist, and the surgeries were conducted by the same team of obstetricians, both of whom were blinded to the group assignments.

At the end of the surgery, patients received an intravenous bolus of either sufentanil (0.1 μg/kg) or hydromorphone (5 μg/kg), followed by PCIA pump connection. The PCIA formulations were: Group S: sufentanil (3 μg/kg) + tropisetron (0.1 mg/kg) in 150 mL saline; Group H: hydromorphone (0.15 mg/kg) + tropisetron (0.1 mg/kg) in 150 mL saline. The concentration for Group H was 0.001 mg/mL (0.15 mg/kg in 150 mL) and for Group S was 0.02 μg/mL (3 μg/kg in 150 mL). Pump settings were: background infusion 2 mL/h, bolus dose 1 mL, lockout interval 15 min, for 48 h. Pumps were prepared by a blinded anesthesia nurse. The specific drug composition and dosage were not disclosed to the study participants.

Postoperative pain management beyond PCIA followed a standardized protocol. No scheduled non-opioid analgesics (e.g., acetaminophen or NSAIDs) were administered. Rescue analgesia with intravenous ketorolac (0.5 mg/kg) was provided only upon patient request and after confirming that two consecutive PCA boluses failed to alleviate resting pain (VAS > 3). Neuraxial morphine was not administered as part of the combined spinal-epidural anesthesia technique. Postoperative follow-up within 48 h was conducted by an anesthesiologist who was also blinded to the group assignments. Furthermore, the data recorder and statistical analyst were also blinded to the group allocations.

### Follow-up procedures and data analysis set

Postoperative follow-up was conducted by a blinded research assistant. For the 48-hour in-hospital period, all assessments (VAS, Ramsay, PSQI, adverse events) were performed at the bedside. For the 1-week and 6-week postpartum assessments, participants were contacted via a pre-scheduled telephone call or a secure video link. A standardized contact protocol was used, with a minimum of three contact attempts (at different times of day and on different days) made before a participant was considered lost to follow-up. To maintain assessor blinding during telephone/video follow-ups, the research assistant followed a script that avoided any reference to the type of analgesia received and instructed participants not to disclose any information about their pain pump experience. All 140 enrolled participants completed the 6-week follow-up, resulting in no missing data for the primary outcome. Consequently, no imputation methods for missing data were required. The primary analysis was performed on the full analysis set, which included all participants who were randomized and received the allocated intervention (modified intention-to-treat), as no patients were excluded from the analysis after intervention initiation.

### Main outcome measures

#### Primary outcome

Incidence of PPD at 6 weeks postoperatively. Diagnosis involved a rigorous two-step process: 1) Screening: All participants completed the Edinburgh Postnatal Depression Scale (EPDS) (Roddy et al., 2026) via a telephone interview or secure online questionnaire, administered by a trained research assistant who was blinded to group allocation. 2) Confirmation: Participants with an EPDS score >9 were referred for a diagnostic confirmation. Within one week, a structured clinical interview based on the Diagnostic and Statistical Manual of Mental Disorders-V (DSM-V) (SCID) (Galbally et al., 2024) was conducted by one of two experienced psychiatrists. These psychiatrists were also blinded to the group allocation and had no access to the patients’ analgesia records or any intra/postoperative data related to pain or opioid use. To prevent unintentional unblinding, participants were explicitly instructed not to discuss their postoperative experience or pain management during the psychiatric interview. Inter-rater reliability for the SCID diagnosis was ensured through regular case conferences and was not formally calculated, but both psychiatrists underwent standardized training on the SCID for this study. The follow-up mode for the SCID was either in-person at the hospital’s psychiatry clinic or via a secure video call, depending on participant preference and availability.

#### Secondary outcomes

Incidence of PPD at 1 week; PSQI scores preoperatively and at 24/48 h; Ramsay scores at 24/48 h; resting VAS scores at 6, 12, 24, 48 h; number of PCIA presses (total/effective), rescue analgesia cases within 48 h, and cumulative 48-h opioid consumption [expressed as oral morphine milligram equivalents (MME)]; bowel function recovery time (the time to pass flatus as the sign of bowel function recovery); first lactation time; neonatal behavioral neurological assessment (NBNA) scores at 48 h postoperatively; adverse reactions within 48 h postoperatively, including postpartum hemorrhage >1,000 mL in 24 h (Madar et al., 2026), nausea/vomiting, dizziness, drowsiness, respiratory depression [SpO_2_ < 90% for 10 s or respiratory rate (RR) < 8 times/min] (Koltenyuk et al., 2024), and pruritus.

#### PSQI scoring criteria

The scale comprises seven components, each scored from 0 to 3, yielding a total score ranging from 0 to 21. While the PSQI was originally developed and validated for a one-month recall period, for the purposes of this acute postoperative assessment at 24 and 48 h, the instructions were verbally modified to ask participants to rate their sleep “over the past 24 h.” We acknowledge that this modified use lacks direct validation, and therefore, postoperative PSQI scores will be treated as continuous, exploratory outcomes reflecting perceived sleep quality, rather than using the standard >5 cutoff to diagnose sleep disorders (Shaowei et al., 2026).

#### Ramsay scoring criteria

Sedation level is scored from 1 to 6: a score of 1 indicates inadequate sedation, scores of 2–4 indicate adequate sedation, and scores of 5 or 6 indicate excessive sedation (Nie et al., 2022).

#### NBNA scoring criteria

The NBNA comprises a total of 20 assessment items, which are divided into five domains: behavioral ability, passive muscle tone, active muscle tone, primary reflexes, and general responses. Each item is scored on a three-point scale (0, 1, or 2), with a maximum total score of 40. Scores ranging from 37 to 40 indicate optimal development, scores of 35–36 warrant comprehensive clinical evaluation, and a score of <35 suggests a risk of neurological impairment (Jiang et al., 2020).

### Statistical analysis

Data analysis was performed using the SPSS 26.0 statistical software package. Normality was assessed with the Shapiro-Wilk test. Normally distributed data are presented as mean ± SD ( $\bar{x}$ ± *s*) and compared using independent t-tests; non-normal data as median (interquartile range) [M (IQR)] using Mann-Whitney U tests. Categorical data are presented as n (%) and compared using χ^2^ or Fisher’s exact test when expected cell counts were <5. For the primary outcome (PPD incidence at 6 weeks), Fisher’s exact test was used. P < 0.05 was considered significant.

For repeatedly measured outcomes (VAS pain scores, PSQI scores, and Ramsay sedation scores), linear mixed-effects models were fitted with group, time, and group-by-time interaction as fixed effects, and participant as a random intercept. An unstructured covariance structure was assumed. Estimated marginal means with 95% CIs are presented.

To control for type I error due to multiple comparisons, a hierarchical testing procedure was prespecified. The primary endpoint (PPD incidence at 6 weeks) was tested first at α = 0.05. Only if this was significant, secondary endpoints were tested in a fixed order: 1) PPD at 1 week, 2) PSQI scores (24 h and 48 h, each tested at α = 0.025 using Bonferroni correction for two time points), 3) Ramsay scores (24 h and 48 h, each at α = 0.025), 4) VAS pain scores (all time points considered as a family, with a global test using mixed model, and post-hoc comparisons at each time point only if the global group × time interaction was significant), and 5) adverse events (each tested at α = 0.05, considered exploratory). All reported P-values for secondary outcomes are nominal and should be interpreted with caution due to the exploratory nature of these analyses.

The primary analysis was performed on a modified intention-to-treat (mITT) population, defined as all randomized participants who received the allocated intervention (i.e., those who completed surgery without intraoperative exclusion criteria). There were no missing data for the primary outcome (PPD at 6 weeks) or for any secondary outcomes among the 137 participants in the mITT analysis set. Therefore, complete-case analysis was effectively performed without the need for imputation.

## Results

### Participant enrollment

**FIGURE 1:**
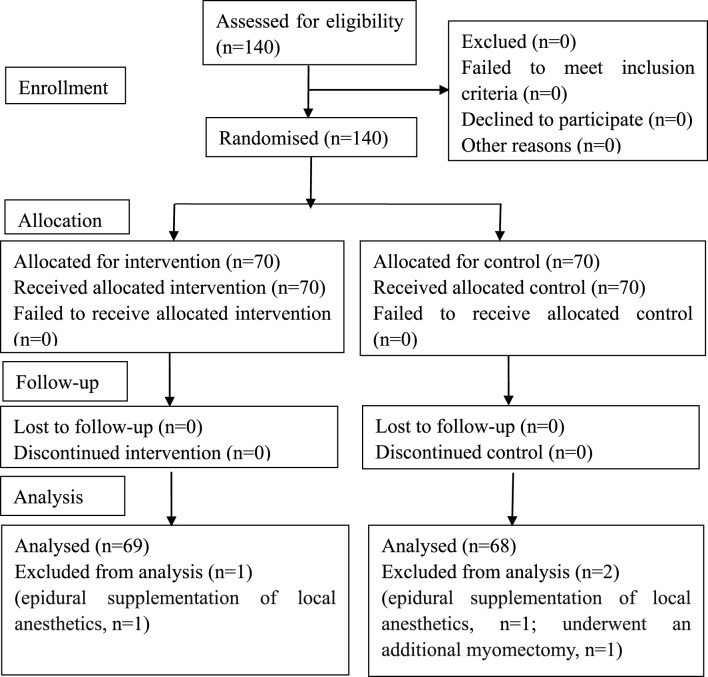
CONSORT flow diagram. In this study, 140 cases were initially enrolled, and 3 cases were excluded. Follow-up for the primary outcome was complete for all 137 participants. In total, data of 69 cases in Group H and 68 cases in Group S were analyzed. CONSORT, Consolidated Standards of Reporting Trials.

A total of 140 participants were randomized. Three participants were excluded after randomization due to intraoperative events (two in Group S: one requiring epidural supplementation, one undergoing additional myomectomy; one in Group H requiring epidural supplementation), leaving 137 participants (69 in Group H, 68 in Group S) in the mITT analysis set. Follow-up for the primary outcome was complete for all 137 participants (Figure 1).

### Demographic data and patient characteristics

No significant differences were found in age, gestational age, BMI, operation time, blood loss, or fluid infusion between groups (Table 1).

**TABLE 1:** Comparison of baseline characteristics in the two groups.

Group	Age (years)	Gestational age (weeks)	BMI (kg/m^2^)	Duration of operation (min)	Intraoperative bleeding loss (mL)	Intraoperative transfusion volume (mL)
Group H (n = 69)	28.2 ± 3.9	38.5 ± 2.7	28.3 ± 4.1	43.5 ± 4.3	308.5 ± 37.3	1361.6 ± 84.7
Group S (n = 68)	27.6 ± 4.7	38.3 ± 3.1	27.4 ± 4.6	41.9 ± 5.2	311 ± 34.9	1325.8 ± 91.3
*P* value	0.842	0.474	0.387	0.347	0.619	0.743

Data are presented as mean ± SD and case numbers (percentage). No significant differences between the two groups (*P* ≥ 0.05); mutual comparison were analyzed using independent-samples t-test; BMI, Body mass index.

### Incidence of PPD and sleep/sedation scores

The incidence of PPD at 6 weeks postoperatively was significantly lower in Group H (4/69, 5.8%) compared to Group S (14/68, 20.6%) (RR = 0.28, 95% CI 0.10–0.78; absolute risk difference = −14.8%, 95% CI −25.4% to −4.2%; NNT = 7, 95% CI 4–24; Fisher’s exact *P* = 0.004) (Table 2).

**TABLE 2:** Incidence of PPD and sleep/sedation scores in the two groups.

Group	PPD		PSQI scores			Ramsay scores	
	1 week	6 weeks	Preoperatively	24 h	48 h	24 h	48 h
Group H (n = 69)	2 (2.9)	4 (5.8)^a^	3.4 ± 1.0	7.3 ± 2.6^a^	6.0 ± 1.8^a^	2.2 ± 0.7^a^	2.0 ± 0.4^a^
Group S (n = 68)	5 (7.3)	14 (20.6)	3.8 ± 0.7	12.2 ± 3.1	10.4 ± 2.6	3.1 ± 1.1	2.7 ± 1.1
*P*-value	0.159	0.004	0.534	0.008	0.006	<0.001	0.009

Data are presented as case numbers (percentage) and mean ± SD. PPD comparisons used Fisher’s exact test; PSQI and Ramsay comparisons used independent t-tests at each time point, with significance evaluated under hierarchical testing (PSQI and Ramsay at 24 h and 48 h each tested at α = 0.025); compared with group S, aP < 0.01. PPD, Postpartum depression; PSQI, Pittsburgh Sleep Quality Index.

Linear mixed-effects models revealed a significant main effect of group on PSQI scores (estimated mean difference [H - S] at 24 h: −4.9, 95% CI −6.2 to −3.6; at 48 h: −4.4, 95% CI −5.7 to −3.1; both *P* < 0.001) and Ramsay scores (24 h difference: −0.9, 95% CI −1.3 to −0.5; 48 h difference: −0.7, 95% CI −1.1 to −0.3; both *P* < 0.01). PSQI and Ramsay scores at 24 and 48 h postoperatively were also significantly lower in Group H (*P* < 0.01) (Table 2).

### Analgesia-related outcomes

For VAS pain scores, the linear mixed model showed no significant group-by-time interaction (*P* = 0.82), indicating similar pain trajectories between groups. Cumulative 48-hour opioid consumption, expressed as oral MME, was similar between groups (Group H: 20.5 ± 1.1 mg vs. Group S: 20.4 ± 1.2 mg, *P* = 0.601), confirming comparable opioid exposure. No significant differences were observed in total/effective PCIA presses, or cases requiring rescue analgesia between the two groups (Table 3).

**TABLE 3:** Analgesia-related profiles in the two groups.

Indicators	Group H (n = 69)	Group S (n = 68)	*P*-value
VAS scores			
6 h	1.3 ± 0.5	1.4 ± 0.4	0.569
12 h	1.8 ± 0.9	2.0 ± 1.1	0.677
24 h	2.2 ± 1.0	2.3 ± 0.9	0.858
48 h	2.4 ± 0.9	2.5 ± 1.2	0.741
PCIA presses			
Total number	4.2 ± 1.3	3.9 ± 1.1	0.542
Effective number	3.5 ± 0.8	3.2 ± 0.9	0.316
Rescue analgesia	4 (5.8)	3 (4.4)	0.663
Cumulative 48-h opioid consumption			
Total volume (mL)	102.5 ± 5.6	101.8 ± 6.1	0.482
Oral MME (mg)	20.5 ± 1.1	20.4 ± 1.2	0.601

Data are presented as mean ± SD and case numbers (percentage). Mutual comparison were analyzed using independent-samples *t*-test and Fisher’s exact test; Linear mixed model showed no significant group-by-time interaction for VAS (*P* = 0.82). VAS, Visual Analog Scale; PCIA, Patient-controlled intravenous analgesia; MME, Morphine milligram equivalent.

### Postoperative adverse reactions

The incidences of dizziness and drowsiness within 48 h postoperatively were significantly lower in Group H compared to Group S (*P* < 0.01). No significant differences were found in bowel function recovery time, first lactation time, NBNA scores at 48 h postoperatively, postpartum hemorrhage, nausea/vomiting, respiratory depression, or pruritus within 48 h postoperatively between the two groups (Table 4).

**TABLE 4:** Postoperative adverse reactions in the two groups.

Indicators	Group H (n = 69)	Group S (n = 68)	*P* value
Postpartum hemorrhage	0 (0)	0 (0)	1.000
Dizziness	3 (4.3)^a^	11 (16.2)	0.007
Drowsiness	1 (1.4)^a^	7 (10.3)	<0.001
Nausea, vomiting	5 (7.2)	4 (5.9)	0.249
Respiratory depression	0 (0)	0 (0)	1.000
Pruritus	2 (2.9)	1 (1.5)	0.718
Bowel function recovery time	​	​	0.627
<24 h	18 (26.1)	16 (23.5)	​
24–48 h	42 (60.9)	39 (57.4)	​
>48 h	9 (13.0)	13 (19.1)	​
First lactation time	​	​	0.319
<24 h	16 (23.2)	14 (20.6)	​
24–48 h	41 (59.4)	38 (55.9)	​
>48 h	12 (17.4)	16 (23.5)	​
NBNA scores (48 h postoperatively)	38 (37–39)	38 (37–39)	0.675

Data presented as case numbers (percentage) and median (interquartile range) [M (IQR)]. Mutual comparison were analyzed using Fisher’s exact test and Mann-Whitney U tests; compared with group S, ^a^ *P* < 0.01. NBNA, Neonatal behavioral neurological assessment.

## Discussion

Hydromorphone is a semi-synthetic opioid analgesic. Its primary mechanism of action involves agonism of μ-opioid receptors within the central nervous system (Meissner et al., 2023). A distinct chemical structure, differing from that of morphine, underlies its analgesic profile (Rodrigues et al., 2021). Furthermore, hydromorphone exhibits relatively stronger affinity for κ- and δ-opioid receptors, which may contribute to its potential advantages in mitigating depressive symptoms and improving sleep disturbances (Manabe et al., 2019). This randomized controlled trial demonstrates that PCIA with hydromorphone following cesarean delivery reduces the incidence of PPD at 6 weeks, improves postoperative sleep quality, and results in lower rates of dizziness and drowsiness compared to sufentanil-based analgesia.

Postpartum depressive mood should be distinguished from clinically diagnosed PPD. The former is typically screened using rating scales such as EPDS, whereas PPD is a psychiatric disorder requiring formal diagnosis through structured clinical interviews (Lyubenova et al., 2021). This study adhered to strict diagnostic criteria for PPD. Parturients initially screened as suspected cases based on EPDS scores underwent further evaluation by psychiatric specialists. According to the DSM-V and WHO guidelines, PPD is defined as a major depressive episode occurring in women without prior psychiatric history, typically emerging within 1–2 weeks after delivery, with symptom severity often peaking at 4–6 weeks. Common features include emotional lability, severe anxiety, panic attacks, and frequent crying (Galbally et al., 2024). Women undergoing cesarean delivery experience unique psychological stressors, potentially exacerbated by surgical trauma and instrumental extraction. Postpartum psychological regression and emotional vulnerability may contribute to a higher incidence of PPD following non-vaginal deliveries compared to vaginal births (Rejnö et al., 2019). Consequently, this study focused on assessing PPD incidence at 1 and 6 weeks post-cesarean. Research by Yang et al. (2023) suggested that PCIA with sufentanil combined with low-dose esketamine may reduce PPD incidence. However, esketamine can stimulate sympathetic activity, potentially increasing the risk of postpartum hemorrhage, and carries additional risks such as hallucinations and nightmares (Wang et al., 2024).

Sufentanil is widely used for postoperative obstetric analgesia due to its potent analgesic efficacy, high therapeutic index, and prolonged duration of action (Nie et al., 2022). Studies by Wang et al. (2022) and Xiao et al. (2025) reported satisfactory analgesia using sufentanil (3 μg/kg) for PCIA or in combination with nalbuphine or esketamine (2 μg/kg) after cesarean section. Based on potency equivalency guidelines from the Association of Anaesthetists and the British Pain Society (El-Boghdadly et al., 2024), the intravenous potency ratio between sufentanil and hydromorphone ranges from approximately 1:50 to 1:75. For safety and comparability, this study employed a 1:50 potency ratio for dose selection: the control group received sufentanil 3 μg/kg for PCIA, compared with hydromorphone 0.15 mg/kg in the intervention group. The similar cumulative 48-hour opioid consumption expressed in oral MME between groups (20.5 ± 1.1 mg vs. 20.4 ± 1.2 mg, *P* = 0.601) confirms that the two groups received clinically comparable opioid exposures, supporting the validity of the equipotent dosing rationale.

Wang et al. (2025) reported that hydromorphone used for PCIA after orthopedic surgery significantly reduced postoperative depression and sleep disturbance scores. Similarly, this study found that hydromorphone-based PCIA after cesarean delivery was associated with a significantly lower incidence of PPD at 6 weeks and lower PSQI scores at 24 and 48 h postoperatively compared to sufentanil. No significant between-group difference in PPD incidence was observed within the first postoperative week, which may reflect the typical clinical course of PPD, wherein symptoms often become more pronounced after the initial week (Galbally et al., 2024). Accumulating evidence suggests that μ-, κ-, and δ-opioid receptors play significant roles in mood regulation and psychological processing (Gefke et al., 2026; Sigunov et al., 2026; Fricker et al., 2025). While μ-opioid receptor agonists provide potent analgesia and sedation, they are also associated with adverse effects such as respiratory depression, constipation, and euphoria (Gefke et al., 2026). In contrast, κ-opioid receptor agonists can produce analgesia while inhibiting addictive behaviors, and combinations of κ-antagonists with δ-agonists exhibit notable anxiolytic and antidepressant properties (Sigunov et al., 2026; Fricker et al., 2025). Beyond its action at μ-receptors, hydromorphone possesses higher affinity for κ- and δ-receptors compared to morphine and sufentanil. While this distinctive receptor activity profile has been proposed as a potential explanation for mood-related benefits (Manabe et al., 2019; Gefke et al., 2026; Sigunov et al., 2026; Fricker et al., 2025; Ershoff, 2023), these pharmacological mechanisms remain speculative and are derived primarily from preclinical studies. Our clinical trial was not designed to test such mechanisms.

An alternative, and perhaps more clinically relevant, explanation lies in the indirect pathways linking improved early recovery to better long-term mental health (Domenicano et al., 2025). Our study found that the hydromorphone group experienced significantly lower rates of dizziness and drowsiness, better early sedation scores (lower Ramsay scores indicating a more alert state), and improved perceived sleep quality (lower PSQI scores) within the first 48 h. These factors collectively contribute to a more positive early postpartum experience. Poor sleep, excessive sedation, and distressing side effects like dizziness are established risk factors for mood disturbances and may impair a new mother’s ability to bond with her infant and engage in early self-care. By mitigating these negative early experiences, hydromorphone may have fostered a more favorable recovery trajectory, thereby reducing the likelihood of developing PPD at 6 weeks. This hypothesis is supported by research linking poor postpartum sleep quality and recovery experience to subsequent depression (Xie et al., 2026; Redhead et al., 2026). Future research should employ mediation analysis to formally test whether improvements in early recovery parameters (e.g., sleep quality, side effect burden) explain the observed reduction in PPD incidence.

In this study, resting VAS scores did not differ significantly between groups, a finding potentially related to the residual neural blockade from combined spinal-epidural anesthesia in the early postoperative period. Research by Nylander et al. (2024) indicated that hydromorphone might exhibit biased signaling via the G protein-coupled pathway of the μ-opioid receptor, potentially yielding improved analgesic efficacy with fewer side effects. Nevertheless, this study found no significant difference in resting VAS scores within 48 h between groups, likely attributable to the equipotent dosing based on established analgesic ratios (Wang et al., 2025). Notably, the incidence of dizziness and drowsiness, along with Ramsay sedation scores within 48 h, were significantly lower in the hydromorphone group. The low and comparable rates of rescue analgesia between groups (5.8% vs. 4.4%, *P* = 0.663) suggest that the observed differences in PPD and sleep outcomes are unlikely to be confounded by differential use of additional analgesics. Furthermore, the results of this study revealed no significant abnormalities or statistical differences between the two groups of parturients in terms of postoperative bowel function recovery time, first lactation time, or NBNA scores at 48 h postoperatively, suggesting the safety of the doses of hydromorphone and sufentanil used in this study.

This study has several limitations. First, it did not assess the severity of PPD or gradations of sleep disturbance. Second, no long-term follow-up was conducted regarding treatment and recovery outcomes for parturients diagnosed with PPD. Third, the use of the PSQI, which is validated for a one-month recall, at 24 and 48 h postoperatively represents a methodological limitation. Although we modified the recall instructions, this approach lacks formal validation and may not fully capture the nuances of acute postoperative sleep disruption. Therefore, our findings related to PSQI scores at these early time points should be considered exploratory and interpreted with caution. Fourth, the strict eligibility criteria [e.g., exclusion of women with pre-existing depressive symptoms (EPDS > 9), poor baseline sleep (PSQI > 5), advanced age, obesity, or medical comorbidities] limit the generalizability of our findings to the broader, higher-risk cesarean population. Therefore, the effect of hydromorphone for PPD in high-risk populations warrants further investigation.

## Conclusion

In summary, hydromorphone-based PCIA after cesarean delivery can reduce the incidence of PPD within 6 weeks postoperatively, alleviate sleep disturbances within the first 48 h, and decrease the occurrence of postoperative dizziness and drowsiness. These findings provide a clinical reference for PPD prevention in parturients following surgical delivery.

## Data Availability

The raw data supporting the conclusions of this article will be made available by the authors, without undue reservation.
